# TNF- **α** augments intratumoural concentrations of doxorubicin in TNF- **α** -based isolated limb perfusion in rat sarcoma models and enhances anti-tumour effects

**DOI:** 10.1054/bjoc.1999.1027

**Published:** 2000-02

**Authors:** A H van der Veen, J H W de Wilt, A M M Eggermont, S T van Tiel, A L B Seynhaeve, T L M ten Hagen

**Affiliations:** Department of Surgical Oncology, University Hospital Rotterdam/Daniel den Hoed Cancer Center, PO Box 5201, Rotterdam, AE, 3008, The Netherlands

**Keywords:** doxorubicin, isolated limb perfusion, rat, sarcoma, tumour necrosis factor-alpha

## Abstract

We have shown previously that isolated limb perfusion (ILP) in sarcoma-bearing rats results in high response rates when melphalan is used in combination with tumour necrosis factor alpha (TNF-α). This is in line with observations in patients. Here we show that ILP with doxorubicin in combination with TNF-α has comparable effects in two different rat sarcoma tumour models. The addition of TNF-α exhibits a synergistic anti-tumour effect, resulting in regression of the tumour in 54% and 100% of the cases for the BN175-fibrosarcoma and the ROS-1 osteosarcoma respectively. The combination is shown to be mandatory for optimal tumour response. The effect of high dose TNF-α on the activity of cytotoxic agents in ILP is still unclear. We investigated possible modes by which TNF-α could modulate the activity of doxorubicin. In both tumour models increased accumulation of doxorubicin in tumour tissue was found: 3.1-fold in the BN175 and 1.8-fold in the ROS-1 sarcoma after ILP with doxorubicin combined with TNF-α in comparison with an ILP with doxorubicin alone. This increase in local drug concentration may explain the synergistic anti-tumour responses after ILP with the combination. In vitro TNF-α fails to augment drug uptake in tumour cells or to increase cytotoxicity of the drug. These findings make it unlikely that TNF-α directly modulates the activity of doxorubicin in vivo. As TNF-α by itself has no or only minimal effect on tumour growth, an increase in local concentrations of chemotherapeutic drugs might well be the main mechanism for the synergistic anti-tumour effects. © 2000 Cancer Research Campaign


*British Journal of Cancer* (2000) **82**(4), 973–980
© 2000 Cancer Research Campaign
DOI: 10.1054/ bjoc.1999.1027, available online at http://www.idealibrary.com on IDEAL®

### TNF-α augments intratumoural concentrations of doxorubicin in TNF-α-based isolated limb perfusion in rat sarcoma models and enhances anti-tumour effects

**AH van der Veen, JHW de Wilt, AMM Eggermont, ST van Tiel, ALB Seynhaeve and TLM ten Hagen**

Department of Surgical Oncology, University Hospital Rotterdam/Daniel den Hoed Cancer Center, PO Box 5201, 3008 AE Rotterdam, The Netherlands

**Summary** We have shown previously that isolated limb perfusion (ILP) in sarcoma-bearing rats results in high response rates when melphalan is used in combination with tumour necrosis factor alpha (TNF-α). This is in line with observations in patients. Here we show that ILP with doxorubicin in combination with TNF-α has comparable effects in two different rat sarcoma tumour models. The addition of TNF-α exhibits a synergistic anti-tumour effect, resulting in regression of the tumour in 54% and 100% of the cases for the BN175-fibrosarcoma and the ROS-1 osteosarcoma respectively. The combination is shown to be mandatory for optimal tumour response. The effect of high dose TNF-α on the activity of cytotoxic agents in ILP is still unclear. We investigated possible modes by which TNF-α could modulate the activity of doxorubicin. In both tumour models increased accumulation of doxorubicin in tumour tissue was found: 3.1-fold in the BN175 and 1.8-fold in the ROS-1 sarcoma after ILP with doxorubicin combined with TNF-α in comparison with an ILP with doxorubicin alone. This increase in local drug concentration may explain the synergistic anti-tumour responses after ILP with the combination. In vitro TNF-α fails to augment drug uptake in tumour cells or to increase cytotoxicity of the drug. These findings make it unlikely that TNF-α directly modulates the activity of doxorubicin in vivo. As TNF-α by itself has no or only minimal effect on tumour growth, an increase in local concentrations of chemotherapeutic drugs might well be the main mechanism for the synergistic anti-tumour effects. © 2000 Cancer Research Campaign

**Keywords:** doxorubicin; isolated limb perfusion; rat; sarcoma; tumour necrosis factor-alpha

Low concentrations at the tumour site and dose-limiting systemic toxicity are common causes for failure of solid tumour treatment with anti-tumour agents. As cytotoxic drugs typically exhibit a steep dose–response curve, increasing local concentration should favour tumour response. In isolated limb perfusions (ILP) local drug concentrations are increased while systemic exposure to the drugs is minimal. In ILP melphalan is used most commonly, but other agents (e.g. doxorubicin and cisplatin) are also applied with varying success in perfusion of limb or organ (e.g. lung) (Tonak et al, 1979; Klaase et al, 1989; Rossi et al, 1992; Weksler et al, 1994; Abolhoda et al, 1997). Tumour necrosis factor alpha (TNF-α), a cytokine with known anti-tumour activity, cannot be used systemically in dosages high enough to obtain a tumour response (Asher et al, 1987; Fajardo et al, 1992). However, in ILP with TNF-α, tumours are exposed to concentrations of up to 50 times higher than those reached after systemic administration of the maximum tolerated dose (MTD), without major side-effects (Benckhuijsen et al, 1988). Previously it was demonstrated that the addition of TNF-α to melphalan in ILP could improve response rates in patients with multiple melanoma in transit metastases or irresectable soft tissue extremity sarcomas (Lienard et al, 1992, 1994; Eggermont et al, 1993, 1996*a*, 1996*b*; Lejeune et al, 1993). In both patient groups very high response rates of above 85% have been reported, with a limb salvage rate of more than 85%. In Europe, TNF-α was recently approved and registered for clinical use in patients for the treatment of locally advanced extremity soft tissue sarcomas by ILP with TNF-α and melphalan. Comparable results have been reported by us for ILP with the combination of TNF-α and melphalan in soft tissue sarcoma and osteosarcoma-bearing rats (Manusama et al, 1996*a*, 1996*b*; de Wilt et al, 1999). ILP with TNF-α alone or melphalan alone at concentrations used in the clinical setting had negligible anti-tumour effects, whereas the combination showed strong synergistic anti-tumour efficacy.

*Received 7 July 1999*
*Revised 27 September 1999*
*Accepted 28 September 1999*

*Correspondence to:* TLM ten Hagen, Department of Experimental Surgical Oncology, Laboratory of Experimental Surgery & Oncology, Erasmus University, Room Ee 0102a, PO Box 1738, 3000 DR Rotterdam, The Netherlands

TNF-α may potentiate the effects of chemotherapy in ILP in various ways. TNF-α has a broad spectrum of activities, which range from enhancement of proliferation to direct cytotoxicity on tumour cells, activation of inflammation and effects on endothelium (Watanabe et al, 1988; Fajardo et al, 1992). The tumour-associated vasculature (TAV) responds to TNF-α with rounding of the endothelial cells resulting in increased gaps, allowing easy passage of soluble materials and even cells (Smyth et al, 1988; Folli et al, 1993; Renard et al, 1995). Moreover, intravenous injection of TNF-α in human melanoma xenograft-bearing mice resulted in significant reduction of the interstitial fluid pressure (IFP) of the tumours (Kristensen et al, 1996). This phenomenon could increase localization of cytotoxic drugs in the tumour interstitium and explain improved tumour response. Secondly, clinical and experimental results demonstrating massive destruction of the endothelial cells, which has also been shown in vitro and on angiograms in patients after ILP, suggest that the TAV is the primary target for TNF-α and therefore that destruction of the endothelial lining might be responsible for the anti-tumour response (Sato et al, 1986; Watanabe et al, 1988; Olieman et al, 1997). This process was accompanied by inflammatory responses and seemed to be dependent on infiltrating leucocytes (Manusama et al, 1998). Coagulative and haemorrhagic necrosis and destruction of the endothelial lining was also seen when TNF-α was used as a single agent in ILP, without significant effect, however, on tumour growth in rats. This indicates that the direct TNF-α effect is most likely playing a minor role in the anti-tumour capacity (Manusama et al, 1996*a*; Nooijen et al, 1996*a*).

Although in the majority of the perfusions, especially for the treatment of melanoma, melphalan is used, other agents might also be successful. Anthracyclines are among the most active agents against solid tumours and doxorubicin is the most widely used agent of this class (Budd, 1995; Bielack et al, 1996). Moreover, doxorubicin is the agent of choice for the treatment of sarcoma, and has shown good anti-tumour activity in clinical and experimental perfusion settings for the treatment of lung metastases (Weksler et al, 1994; Abolhoda et al, 1997), and could therefore be a suitable cytotoxic agent for ILP in sarcoma-bearing patients.

In this study we undertook ILP with doxorubicin and TNF-α in soft tissue sarcoma- and osteosarcoma-bearing rats to examine the effect of TNF-α on the anti-tumour activity of doxorubicin. Secondly, an attempt was made to unravel possible mechanisms by which TNF-α potentiates the anti-tumour activity of doxorubicin.

### MATERIALS AND METHODS

### Chemicals

Human recombinant TNF-α (specific activity $5 \times 10^7$ IU mg$^{-1}$) was kindly provided by Dr G Adolf (Bender Wien GmbH, Wien, Austria) and stored at a concentration of 2 mg ml$^{-1}$ at –80°C or under liquid nitrogen. Endotoxin levels (LAL) were below 0.624 EU mg$^{-1}$. Doxorubicin (Adriablastina®) was purchased from Farmitalia Carlo Erba (Brussels, Belgium).

### Animals and tumour model

Male inbred BN rats were used for the soft tissue sarcoma model (BN175) and WAG/RIJ rats for the osteosarcoma model (ROS-1). Rats were obtained from Harlan-CPB (Austerlitz, The Netherlands) and weighed 250–300 g. Small fragments (3 mm) of the syngeneic BN175 or ROS-1 sarcoma were implanted subcutaneously in the right hindleg as previously described (Manusama et al, 1996*a*; 1996*b*). Tumour growth was recorded by calliper measurements and tumour volume calculated using the formula $0.4(A^2 \times B)$ (where B represents the largest diameter and A the diameter perpendicular to B). All animal studies were done in accordance with protocols approved by the Animal Care Committee of the Erasmus University Rotterdam, The Netherlands.

The classification of tumour response was: progressive disease (PD), increase of tumour volume (> 25%) within 5 days; no change (NC), tumour volume equal to volume during perfusion in a range of –25% and + 25%; partial remission (PR), decrease of tumour volume between –25% and –90%; complete remission (CR), tumour volume less than 10% of initial volume.

### ILP protocol

Rats were perfused according to the ILP technique originally described by Benckhuijsen et al (1982*a*) and adapted for the rat by Manusama et al (Manusama et al, 1996*a*). Briefly, the femoral artery and vein of anaesthetized rats were cannulated with silastic tubing. Collaterals were occluded by a groin tourniquet and perfusion started when the tourniquet was tightened. The extracorporeal circuit included an oxygenation reservoir and a roller pump (Watson Marlow, Falmouth, UK). The perfusion was performed with 5 ml Haemaccel (Behring Pharma, Amsterdam, The Netherlands) and TNF-α (50 μg) and/or doxorubicin (400 μg BN175 and 200 μg ROS-1) were added as boluses to the oxygenation reservoir. Control rats (sham) were perfused with Haemaccel alone. The concentration of TNF-α was adapted from previous animal studies and doxorubicin concentrations chosen had no local toxicity and induced maximally stable disease after single perfusion (Manusama et al, 1996*a*). ILP with lower doxorubicin dosages or TNF dosages below 10 μg resulted in comparable or diminished tumour response (de Wilt et al, 1999). Perfusion was maintained for 30 min at a flow rate of 2.4 ml min$^{-1}$. During the perfusion the hindleg of the rat was kept at a temperature of 38–39°C with a warm water mattress. A washout with 2 ml oxygenated Haemaccel was performed at the end of the perfusion. Perfusion was performed at a tumour diameter of 12–15 mm, which is around 7 or 10 days after implantation for BN-175 and ROS-1 respectively.

### In vitro assessment of anti-tumour activity

BN175 soft tissue sarcoma cells or ROS-1 osteosarcoma cells were added in 100 μl aliquots to 96-well plates at a final concentration of $10^4$ cells per well and allowed to grow as a monolayer in Dulbecco’s modified Eagle’s medium (DMEM) supplemented with 10% fetal calf serum (FCS). Doxorubicin and/or TNF-α were diluted in DMEM supplemented with 10% FCS, added to the wells and allowed to incubate for 3 days. The range of final drugs in the wells was 0.0005–100 μg ml$^{-1}$ for doxorubicin and 0–10 μg ml$^{-1}$ for TNF-α. A total of 5–6 separate assays were performed in triplicate and the percentage of growth inhibition calculated according to the formula: percentage of tumour cell growth = (test well/control well) × 100%. Percentage of tumour cell cytotoxicity was measured using the sulphorhodamine B assay (Keepers et al, 1991).

### In vitro assessment of doxorubicin uptake in tumour cells

To determine if the observed anti-tumour response after ILP and cytotoxicity in vitro correlated with cellular uptake of doxorubicin, cells were exposed to doxorubicin with and without TNF-α and intracellular doxorubicin levels determined by flow cytometry as previously described (Luk and Tannock, 1989). Briefly, BN175 soft tissue sarcoma cells or ROS-1 osteosarcoma cells were added in 500-μl aliquots to 24-well plates at a final concentration of $5 \times 10^4$ cells per well and allowed to grow as a monolayer in DMEM supplemented with 10% FCS. Doxorubicin and TNF-α were diluted in DMEM supplemented with 10% FCS and added to the wells, after which cells were incubated for 0, 10, 30, 60 and 120 min. The final drug concentration in the wells was

© 2000 Cancer Research Campaign

0, 0.1, 1.0 and 10 μg ml$^{-1}$ for both doxorubicin and TNF-α. Thereafter monolayers were treated with trypsin–EDTA for 2 min and the cell suspensions were washed two times in complete medium and resuspended in phosphate-buffered saline (PBS). Cellular uptake was measured on a Becton Dickinson FACScan using Cell Quest software on Apple Macintosh computer. Excitation was set at 488 nm and emission at 530 nm. Fluorescence was corrected for cell size using the forward scatter (FSC) with the formula corrected fluorescence (FLcor) = fluorescence at 530 nm (FL530)/FSC-FL530$_c$/FSC$_c$ (FL530$_c$ and FSC$_c$ are fluorescence and forward scatter with no drug added to the cells).

### Assessment of doxorubicin accumulation in solid tumour and concentration in perfusate during ILP

To determine the influence of TNF-α on doxorubicin accumulation in tumours during ILP, tumours (and muscle) were surgically removed after ILP and total doxorubicin content determined as previously described (Mayer et al, 1989). As the ILP included a thorough washout there was no intravascular doxorubicin present. Briefly, after incubation in acidified isopropanol (0.075 N hydrochloric acid in 90% isopropanol) for 24 h at 4°C, the tumours were homogenized (PRO200 homogenizer with 10-mm generator, Pro Scientific, CT, USA), centrifuged for 30 min at 2500 rpm and supernatants harvested. Samples were measured in a Hitachi F4500 fluorescence spectrometer (excitation 472 nm and emission 590 nm) and compared with a standard curve prepared with known concentrations of doxorubicin diluted in acidified isopropanol. Measurements were repeated after addition of an internal doxorubicin standard. Detection limit for doxorubicin in tissue was 0.1 μg g$^{-1}$ tissue.

For perfusate measurements samples were drawn from the perfusion vial at 0.5, 5, 15 and 30 min after ILP was started. Samples were centrifuged for 30 min at 2500 rpm and supernatant measured for doxorubicin content as described above. Cell pellets were incubated in acidified isopropanol and doxorubicin content determined as described above.

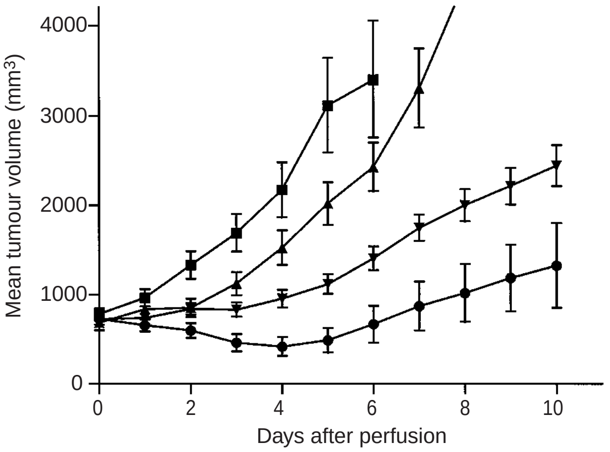

**Figure 1** Growth curves of subcutaneous implanted soft tissue sarcoma BN175 after isolated limb perfusion with medium alone (■), 50 μg TNF-α (▲), 400 μg doxorubicin (▼), or combination of TNF-α and doxorubicin (●). Mean tumour volumes are shown ± s.e.m. Number of rats per group is shown in Table 1

### Statistical analysis

The in vivo and in vitro results were evaluated for statistical significance using the Mann–Whitney *U*-test with SPSS for windows. In vitro data were analysed by curve fitting using GraphPad Prism. *P*-values below 0.05 were considered statistically significant.

### RESULTS

### In vivo tumour response to doxorubicin and TNF-α

To evaluate the anti-tumour activity of doxorubicin when combined with TNF-α in an ILP setting, soft tissue sarcoma and osteosarcoma-bearing rats were perfused with the agents combined or alone. Figure 1 shows the tumour responses of soft tissue sarcoma (BN175) in rats after ILP. Perfusion with buffer or TNF-α alone resulted in progressive disease in all animals. Although ILP with doxorubicin (400 μg) alone resulted in a slight inhibition of the BN175 tumour growth when compared with the sham control, none of the rats showed a tumour response (Table 1). ILP with 400 μg doxorubicin combined with 50 μg TNF-α resulted in increased anti-tumour activity with a response rate of 54% (PR and CR combined; $P < 0.01$ compared with doxorubicin alone).

In osteosarcoma (ROS1)-bearing rats ILP with buffer or doxorubicin (200 μg) alone had no significant effect on tumour growth (Figure 2). ILP with TNF-α alone resulted in significant inhibition of tumour growth as compared with the sham perfusion

**Table 1** Tumour response of BN-175 after ILP with doxorubicin and TNF-α during 5 days after treatment

| Response[a] | Sham *n* = 12 | TNF-α[b] *n* = 10 | Doxorubicin *n* = 10 | Doxorubicin + TNF-α *n* = 13 |
|---|---|---|---|---|
| PD | 12 | 10 | 6 | 2 |
| NC | | | 4 | 4 |
| PR | | | | 6 |
| CR | | | | 1 |
| Response rate (PR and CR) | | | | 54% |

[a]Responses were scored as described in Materials and Methods. [b]TNF-α and doxorubicin, 50 and 400 μg respectively, were added to the perfusate (5 ml) as boluses. [c]PD: progressive disease, NC: no change, PR: partial remission, CR: complete remission.

**Table 2** Tumour response of ROS-1 after ILP with doxorubicin and TNF-α during 5 days after treatment

| Response[a] | Sham *n* = 8 | TNF-α[b] *n* = 9 | Doxorubicin *n* = 8 | Doxorubicin + TNF-α *n* = 10 |
|---|---|---|---|---|
| PD | 8 | 3 | 2 | |
| NC | | 3 | 6 | |
| PR | | 1 | | 6 |
| CR | | 2 | | 4 |
| Response rate (PR and CR) | | 33% | | 100% |

[a]Classification of response rates is described in Materials and Methods. [b]TNF-α and doxorubicin, 50 and 200 μg respectively, were added to the perfusate (5 ml) as boluses. PD: progressive disease, NC: no change, PR: partial remission, CR: complete remission.

© 2000 Cancer Research Campaign

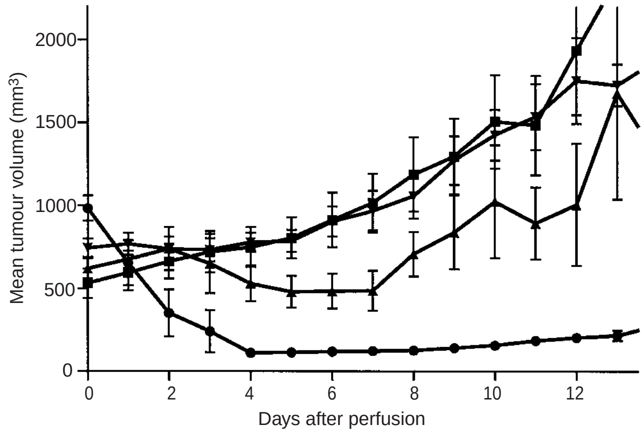

**Figure 2** Growth curves of subcutaneous implanted osteosarcoma ROS-1 after isolated limb perfusion with medium alone (■), 50 µg TNF-α (▲), 200 µg doxorubicin (▼), or combination of TNF-α and doxorubicin (●). Mean tumour volumes are shown ± s.e.m. Number of rats per group is shown in Table 2

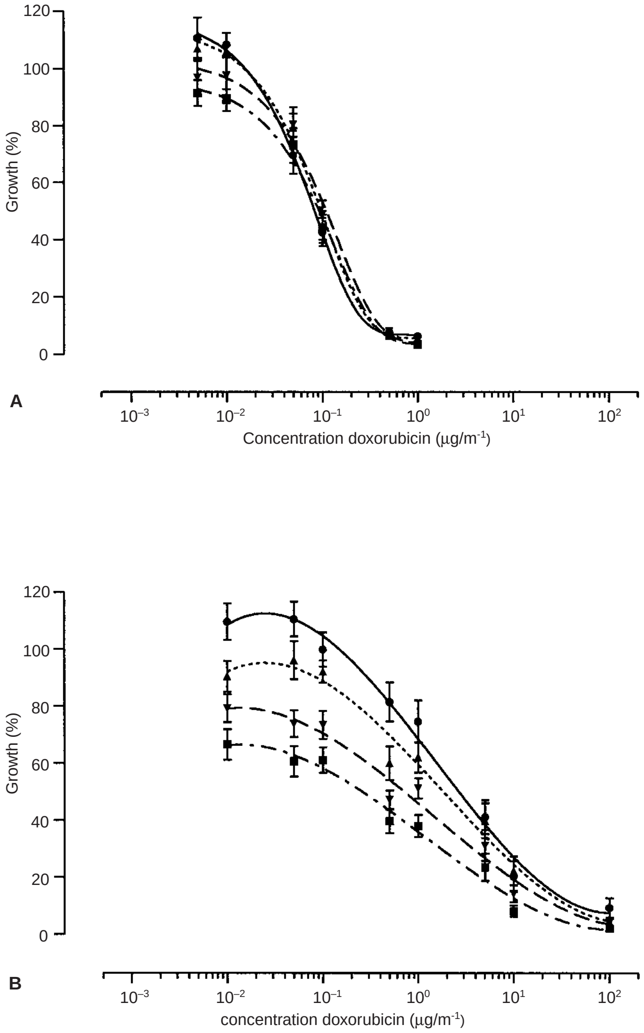

**Figure 3** In vitro growth of (**A**) BN175 and (**B**) ROS-1 tumour cells as function of the doxorubicin concentration in combination with 0 µg (●), 0.1 µg (▲), 1.0 µg (▼) or 10 µg TNF-α per ml (■). The mean of 5–6 individual experiments performed in triple is shown ± s.e.m.

and a response rate of 33% was observed (Table 2). ILP with 200 µg doxorubicin combined with 50 µg TNF-α further increased the anti-tumour response with a response rate of 100% (PR and CR combined; $P < 0.05$ compared with TNF-α alone).

### In vitro assessment of anti-tumour activity of doxorubicin and TNF-α

The in vivo experiments clearly demonstrate pronounced improvement of tumour response when doxorubicin was used in combination with TNF-α. In vitro experiments were performed to further study the nature of this interaction. Exposure of soft tissue sarcoma BN175 or osteosarcoma ROS-1 tumour cells to doxorubicin resulted in a response curve with an $IC_{50}$ of 0.1 and 2.0 µg $ml^{-1}$ respectively (Figure 3). No significant cellular toxicity could be observed when BN175 cells were exposed to TNF-α alone; however, a dose-dependent growth reduction was observed when ROS-1 cells were exposed to TNF-α with a maximum reduction of 38% at 10 µg $ml^{-1}$. Addition of TNF-α to doxorubicin did not significantly alter the $IC_{50}$ of doxorubicin in the BN-175 cell cultures, indicating that addition of TNF-α in vitro did not influence the sensitivity of the cells to doxorubicin significantly. On ROS-1 cells only an additive effect of TNF-α and doxorubicin was observed. The curve only shifted downwards and not to a lower doxorubicin concentration, which indicates that the drugs do not influence each other but have separate effects.

### In vitro uptake of doxorubicin in tumour cells

Figure 4 shows that increased intracellular concentrations of doxorubicin are observed in both cell types when cells were incubated with increasing concentrations of doxorubicin. A tenfold higher doxorubicin concentration in culture supernatant (ranging from 1.0 to 10 µg $ml^{-1}$) resulted in 4.5-fold and 3.9-fold augmented cellular uptake for BN175 and ROS-1 respectively ($P < 0.01$ and $P < 0.05$). Addition, however, of TNF-α to the culture medium did not influence intracellular doxorubicin content significantly for all the TNF-α concentrations tested, or even a slight but not significant reduction in uptake was noticed with increasing concentrations of TNF-α (Figure 5).

### Doxorubicin accumulation in solid tumour after ILP

Possibly the observed beneficial effect of TNF-α in vivo could be explained by an increased extravasation of doxorubicin into the tumour interstitium, resulting in a higher local concentration and accordingly in an improved anti-tumour activity. Therefore, concentrations of doxorubicin in tumour and surrounded tissue after ILP were determined. Figure 6 shows that measurable amounts of doxorubicin localized both in BN175 and ROS-1 tumours after ILP, which correlates with an observed decline of the drug concentration in the perfusate (data not shown). Moreover, addition of TNF-α to the perfusate resulted in significantly enhanced accumulation of doxorubicin in both these tumours, 3.1-fold in the BN175 and 1.8-fold in the ROS-1 sarcoma, when compared with ILP with doxorubicin alone. Addition of TNF had no significant effect on doxorubicin accumulation in muscle of the leg ($P > 0.4$). Strikingly, a significant discrepancy in drug levels was observed between BN175 and ROS-1 tumours.

 © 2000 Cancer Research Campaign

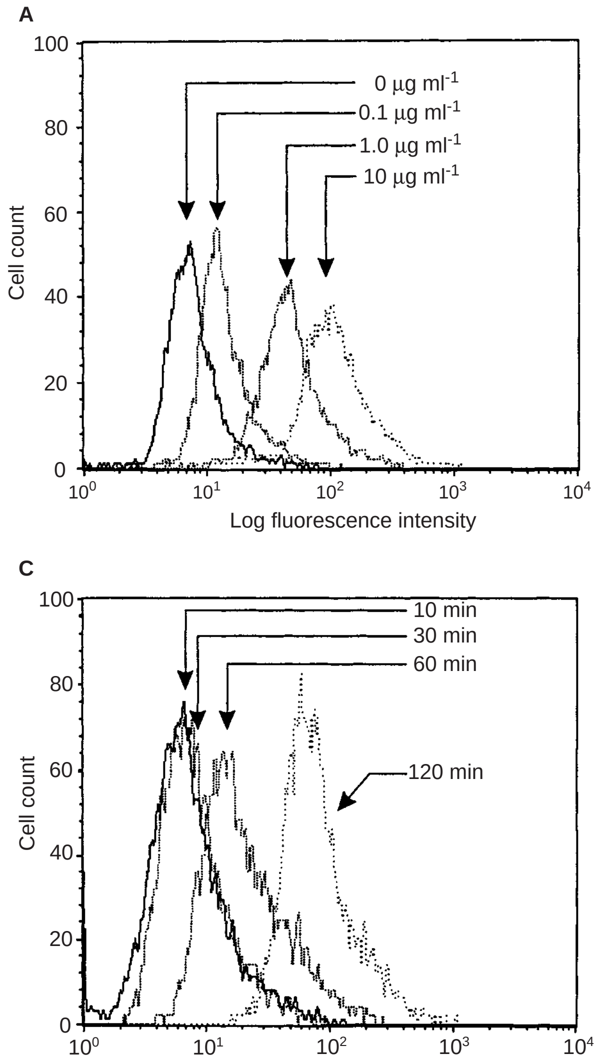

**Figure 4** Uptake of doxorubicin by (**A** and **C**) BN175 tumour cells, or (**B** and **D**) ROS-1 tumour cells in vitro as determined by flowcytometry after exposure of the cells to 0, 0.1, 1.0 or 10 μg ml$^{-1}$ doxorubicin for 2 h (**A** and **B**) or for various durations of time at a fixed doxorubicin concentration of 10 μg ml$^{-1}$ (**C** and **D**). The graphs are good representatives of the experiments performed

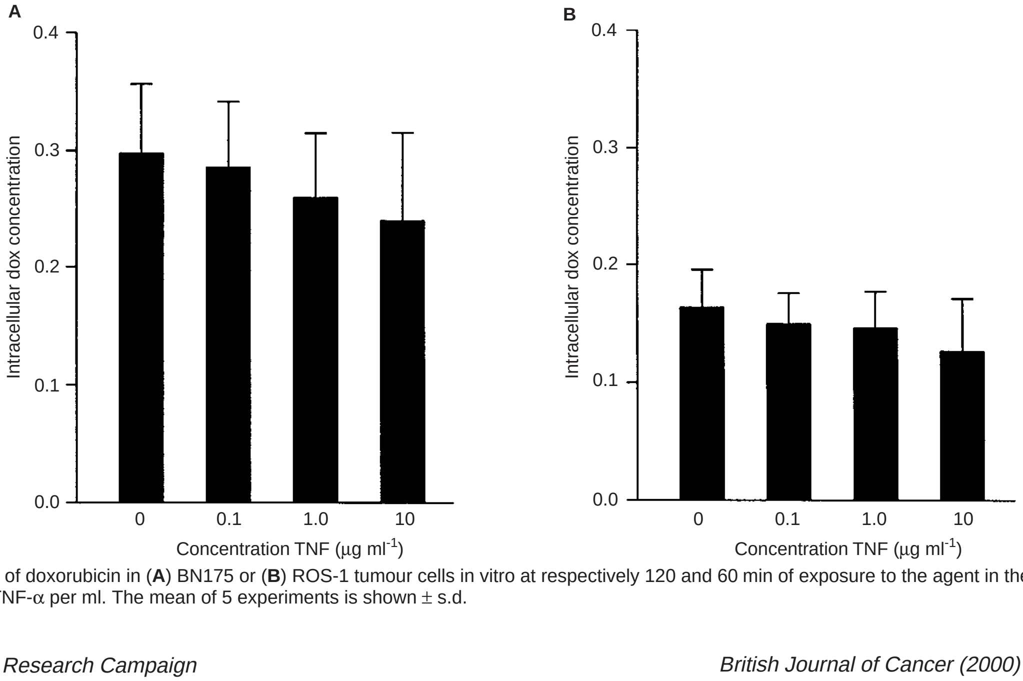

**Figure 5** Uptake of doxorubicin in (**A**) BN175 or (**B**) ROS-1 tumour cells in vitro at respectively 120 and 60 min of exposure to the agent in the presence of 0, 0.1, 1.0 or 10 μg TNF-α per ml. The mean of 5 experiments is shown ± s.d.

*© 2000 Cancer Research Campaign*

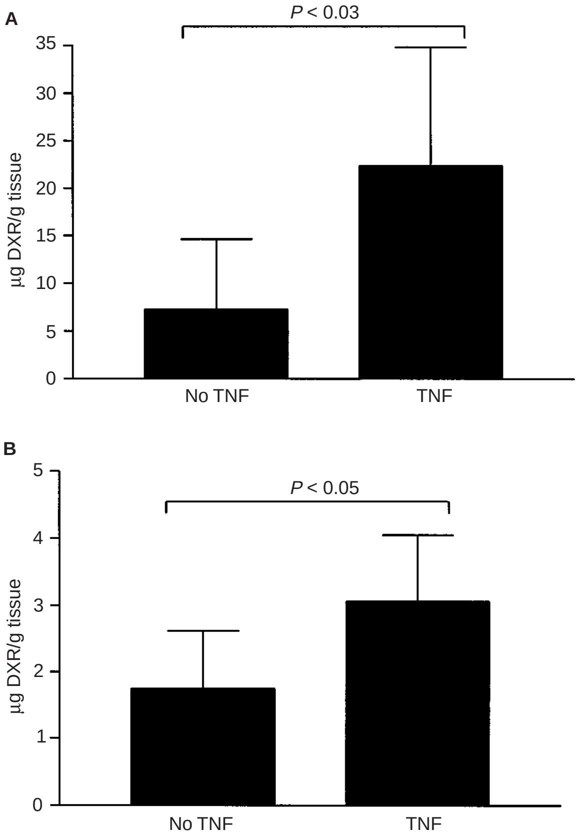

**Figure 6** Accumulation of doxorubicin in (**A**) soft tissue sarcoma BN175 or (**B**) osteosarcoma ROS-1 in vivo during isolated perfusion. Rats were perfused with doxorubicin (400 µg BN175 and 200 µg ROS-1) with 50 µg TNF-α or without TNF-α, after which tumours and muscle were excised and total doxorubicin content determined as described in Materials and Methods. The mean of 6 rats is shown ± s.d.

### DISCUSSION

In the present study we demonstrate that ILP in sarcoma-bearing rats with doxorubicin in combination with TNF-α results in high response rates in two different tumour models. These findings are in close agreement with our previous work using melphalan (Manusama et al, 1996*a*, 1996*b*). Secondly, it is demonstrated for the first time that TNF-α enhances intratumoural accumulation of doxorubicin, which is an attractive explanation for the augmented tumour response in TNF-α-based ILP. We speculate that TNF-α increases interstitial drug levels in the tumour as intravascular doxorubicin is washed out at the end of the ILP procedure and intracellular uptake of doxorubicin is not affected by TNF-α as was shown in vitro.

Doxorubicin has been shown to be the most effective drug in treatment of sarcomas and therefore put forward as the drug of choice in the treatment of these malignancies (Budd, 1995; Bielack et al, 1996). Here we demonstrate that perfusion with doxorubicin alone is not, or only partially, effective, which is also observed when melphalan is used as a single agent in the perfusion setting.

A striking observation is the augmentation of the doxorubicin-induced anti-tumour response by TNF-α in vivo, which has also been shown for melphalan and TNF-α in these tumour models (Manusama et al, 1996*a*, 1996*b*). Strong tumour responses were observed in both models after ILP with the combination therapy, which cannot be explained by just adding up the responses after ILP with the single agents. An important observation is that chemotherapy by itself is not, or partially, effective as shown here and by others (Klaase et al, 1989). Secondly, it was previously shown in our rat tumour model as well as in the clinic that ILP with TNF-α alone had no effect on tumour growth although massive haemorrhagic necrosis and pathology was observed (Posner et al, 1994; Manusama et al, 1996*a*; Nooijen et al, 1996). These observations indicate that other mechanisms have to be identified to explain the interaction between TNF-α and chemotherapy.

Several specific activities of TNF-α could potentiate the anti-tumour activity of chemotherapy. It has been postulated that the increased tumour response observed after ILP with melphalan and TNF-α is due to destruction of the TAV, resulting in haemorrhagic necrosis, platelet aggregation and erythrostasis (Watanabe et al, 1988; Nooijen et al, 1996). Moreover, it has been shown recently that perfusion with melphalan in combination with TNF-α and interferon gamma (IFN-γ) resulted in apoptosis of endothelial cells of the TAV (Ruegg et al, 1998). Inflammatory events such as granulocyte infiltration were also suggested to play a role (Nooijen et al, 1996; Manusama et al, 1998). These findings led to the speculation that destruction of the TAV is the mechanism by which TNF-α potentiates cytotoxic agents. Watanabe et al demonstrated toxic effects of TNF-α on newly formed tumour vasculature in mice, resulting in haemorrhage, congestion and blood circulation blockage (Watanabe et al, 1988). Congestion and blockage of the blood circulation could result in impaired drainage of the tumour leading to the observed augmented tumour concentrations. Others suggested that TNF-α-induced thrombus formation played an important role (Shimomura et al, 1988). However, these effects are also observed after perfusion with TNF-α alone (Nooijen et al, 1996).

Recent studies show that perfusion of melanoma-bearing patients with melphalan in combination with TNF-α and IFN-γ results in detachment and apoptosis of endothelial cells of the tumour (Ruegg et al, 1998). Moreover, the in vitro experiments demonstrated an important role for TNF-α and IFN-γ mediated down-modulation of the $\alpha_v\beta_3$ function, which is speculated to play a prominent role in the in vivo observations. These findings would argue in favour for a TNF-α-mediated destruction of the vasculature. The in vitro observations also demonstrated the necessity of IFN-γ for the induction of endothelial apoptosis. On the other hand it has been shown in our model, as well as in various clinical trails, that tumour responses are only slightly improved by the addition of IFN-γ (Eggermont et al, 1996*b*; Lienard et al, 1992*b*). This would argue against an important role for TNF-α-mediated destruction of the TAV in the tumour response; it also indicates that endogenous produced IFN-γ is of major importance.

A consistent finding in our two models is the augmented accumulation of doxorubicin in tumour tissue when TNF-α is added to the perfusate. In both models this increase could very well explain the improved efficacy. On the other hand, TNF-α may increase the uptake of doxorubicin by the tumour cells. However, intracellular concentration of doxorubicin in vitro was not enhanced when TNF-α was added in vitro. Moreover, TNF-α did not seem to affect the in vitro cytotoxic activity of doxorubicin significantly. In contradiction to these findings, synergy between TNF-α and

*© 2000 Cancer Research Campaign*

doxorubicin in vitro has been shown in previous studies depending on sensitivity of the cells to TNF-α, presence of multidrug resistance or order of exposure (Alexander et al, 1987; Bonavida et al, 1990; Soranzo et al, 1990; Fruehauf et al, 1991). This effect has also been shown without an increased intracellular accumulation of doxorubicin (Safrit et al, 1993). Others demonstrated that exposure of tumour cells to TNF-α resulted in a reduced sensitivity of these cells to doxorubicin (Prewitt et al, 1994). It is suggested that arrest of the cells in the G1/0 phase by TNF-α turns them insensitive to doxorubicin, which is a cell cycle-dependent cytotoxic agent. In our study we did not observe such phenomenon when the tumour cells were exposed to doxorubicin and TNF-α. These observations suggest that in vivo TNF-α has an indirect effect on the anti-tumour activity of doxorubicin. Therefore, we postulate that TNF-α augments the accumulation of doxorubicin in the tumour by increasing the leakiness of the TAV, and by doing so increases the local drug level. Previously an increased leakiness of the TAV as well as a reduction of the IFP in tumour has been shown by others after systemic administration of TNF-α (Smyth et al, 1988; Folli et al, 1993; Renard et al, 1995; Kristensen et al, 1996). In patients and in other experimental models, accumulation of doxorubicin in tumour was found to fluctuate between 1.0 and 7.0 $\mu g\ g^{-1}$ tissue after intravenous treatment, but considerably higher when locally infused (around 20 $\mu g\ g^{-1}$ tumour), which is comparable with our findings when ILP is performed with doxorubicin alone (Lee et al, 1980; Ridge et al, 1988; Murdter et al, 1997). Moreover, increased drug accumulation in tumour has previously been shown after systemic treatment with TNF-α when a liposomal doxorubicin preparation was injected (Suzuki et al, 1990). It must be kept in mind that accumulation of drug in tumour not only depends on treatment procedure but also on the type of tumour. Preliminary results from a clinical phase I–II trial with doxorubicin and TNF-α in hyperthermic ILP demonstrated comparable favourable outcome as is obtained with melphalan and TNF-α (Di Filippo et al, 1998).

From our study we propose that the observed augmentation of the anti-tumour activity of doxorubicin by TNF-α is mainly due to an increased accumulation of doxorubicin in the tumour during ILP as is shown in both models. A direct effect of TNF-α on the sensitivity of the tumour cells to doxorubicin was ruled out by in vitro examinations.

### REFERENCES

Abolhoda A, Brooks A, Nawata S, Kaneda Y, Cheng H and Burt ME (1997) Isolated lung perfusion with doxorubicin prolongs survival in a rodent model of pulmonary metastases. *Ann Thorac Surg* **64:** 181–184

Alexander RB, Nelson WG and Coffey DS (1987) Synergistic enhancement by tumor necrosis factor of in vitro cytotoxicity from chemotherapeutic drugs targeted at DNA topoisomerase II. *Cancer Res* **47:** 2403–2406

Asher A, Mule JJ, Reichert CM, Shiloni E and Rosenberg SA (1987) Studies on the anti-tumor efficacy of systemically administered recombinant tumor necrosis factor against several murine tumors in vivo. *J Immunol* **138:** 963–974

Benckhuijsen C, Kroon BB, Van Geel AN and Wieberdink J (1988) Regional perfusion treatment with melphalan for melanoma in a limb: an evaluation of drug kinetics. *Eur J Surg Oncol* **14:** 157–163

Benckhuijsen C, van Dijk WJ and Van't Hoff SC (1982) High-flow isolation perfusion of the rat hind limb in vivo. *J Surg Oncol* **21:** 249–257

Bielack SS, Erttmann R, Kempf-Bielack B and Winkler K (1996) Impact of scheduling on toxicity and clinical efficacy of doxorubicin: what do we know in the mid-nineties? *Eur J Cancer* **32A:** 1652–1660

Bonavida B, Tsuchitani T, Zighelboim J and Berek JS (1990) Synergy is documented in vitro with low-dose recombinant tumor necrosis factor, cisplatin, and doxorubicin in ovarian cancer cells. *Gynecol Oncol* **38:** 333–339

Budd GT (1995) Palliative chemotherapy of adult soft tissue sarcomas. *Semin Oncol* **22:** 30–34

de Wilt JH, Manusama ER, van Tiel ST, Van Ijken MG, ten Hagen TL and Eggermont AM (1999) Prerequisites for effective isolated limb perfusion using tumour necrosis factor alpha and melphalan in rats. *Br J Cancer* **80:** 161–166

Di Filippo F, Vaglini M, Azzarelli A, Anza M, Garinei R, Cavaliere F, Deraco M, Giannarelli D, Vecchiato A, Quagliolo V and Cavaliere R (1998) Hyperthermic antiblastic perfusion with tumor necrosis factor and doxorubicin for the treatment of soft tissue limb sarcoma in candidates for amputation: results of a phase I–II study. Eur J Surg Oncol **24:** 323 (abstract)

Eggermont AMM, Lienard D, Schraffordt Koops H, et al (1993) Treatment of irresectable soft tissue sarcomas of the limbs by isolation perfusion with high dose TNF-alpha in combination with interferon-gamma and melphalan. In: *Tumor Necrosis Factor: Molecular and Cellular Biology and Clinical Relevance*, Fiers W and Buurman WA (eds), pp. 239–243. Karger: Basel

Eggermont AMM, Schraffordt Koops H, Klausner JM, Kroon BBR, Schlag PM, Lienard D, Van Geel AN, Hoekstra HJ, Meller I, Nieweg OE, Kettelhack C, Ben-Ari G, Pector J-C and Lejeune FJ (1996*a*) Isolated limb perfusion with tumor necrosis factor and melphalan for limb salvage in 186 patients with locally advanced soft tissue extremity sarcomas. The cumulative multicenter European experience. *Ann Surg* **224**: 764–765.

Eggermont AMM, Schraffordt Koops H, Lienard D, Kroon BBR, Van Geel AN, Hoekstra HJ, and Lejeune FJ (1996*b*) Isolated limb perfusion with high-dose tumor necrosis factor-alpha in combination with interferon-gamma and melphalan for non-resectable extremity soft tissue sarcomas: a multicenter trial. *J Clin Oncol* **14**: 2653–2665

Fajardo LF, Kwan HH, Kowalski J, Prionas SD and Allison AC (1992) Dual role of tumor necrosis factor-alpha in angiogenesis. *Am J Pathol* **140**: 539–544

Folli S, Pelegrin A, Chalandon Y, Yao X, Buchegger F, Lienard DX, Lejeune F and Mach JP (1993) Tumor-necrosis factor can enhance radio-antibody uptake in human colon carcinoma xenografts by increasing vascular permeability. *Int J Cancer* **53**: 829–836

Fruehauf JP, Mimnaugh EG and Sinha BK (1991) Doxorubicin-induced cross-resistance to tumor necrosis factor (TNF) related to differential TNF processing. *J Immunother* **10**: 165–173

Keepers YP, Pizao PE, Peters GJ, van Ark-Otte J, Winograd B and Pinedo HM (1991) Comparison of the sulforhodamine B protein and tetrazolium (MTT) assays for in vitro chemosensitivity testing. *Eur J Cancer* **27**: 8
97–900

Klaase JM, Kroon BB, Benckhuijsen C, Van Geel AN, Albus-Lutter CE and Wieberdink J (1989) Results of regional isolation perfusion with cytostatics in patients with soft tissue tumors of the extremities. *Cancer* **64**: 616–621

Kristensen CA, Nozue M, Boucher Y and Jain RK (1996) Reduction of interstitial fluid pressure after TNF-alpha treatment of three human melanoma xenografts. *Br J Cancer* **74**: 533–536

Lee YT, Chan KK, Harris PA and Cohen JL (1980) Distribution of adriamycin in cancer patients: tissue uptakes, plasma concentration after IV and hepatic IA administration. *Cancer* **45**: 2231–2239

Lejeune FJ, Lienard D, Leyvraz S and Mirimanoff RO (1993) Regional therapy of melanoma. *Eur J Cancer* **29A**: 606–612

Lienard D, Eggermont AMM, Schraffordt Koops H, Kroon BBR, Rosenkaimer F, Autier P and Lejeune FJ (1994) Isolated perfusion of the limb with high-dose tumour necrosis factor-alpha (TNF-alpha), interferon-gamma (IFN-gamma) and melphalan for melanoma stage III. Results of a multi-centre pilot study. *Melanoma Res* **4**: 21–26

Lienard D, Ewalenko P, Delmotte JJ, Renard N and Lejeune FJ (1992) High-dose recombinant tumor necrosis factor alpha in combination with interferon gamma and melphalan in isolation perfusion of the limbs for melanoma and sarcoma. *J Clin Oncol* **10**: 52–60

Luk CK and Tannock IF (1989) Flow cytometric analysis of doxorubicin accumulation in cells from human and rodent cell lines. *J Natl Cancer Inst* **81**: 55–59

Manusama ER, Nooijen PT, Stavast J, de Wilt JH, Marquet RL and Eggermont AM (1998) Assessment of the role of neutrophils on the antitumor effect of TNF-alpha in an in vivo isolated limb perfusion model in sarcoma-bearing brown Norway rats. *J Surg Res* **78**: 169–175

Manusama ER, Nooijen PTGA, Stavast J, Durante NMC, Marquet RL and Eggermont AMM (1996*a*) Synergistic antitumour effect of recombinant human tumour necrosis factor alpha with melphalan in isolated limb perfusion in the rat. *Br J Surg* **83**: 551–555

Manusama ER, Stavast J, Durante NMC, Marquet RL and Eggermont AMM (1996*b*) Isolated limb perfusion with TNF alpha and melphalan in a rat osteosarcoma model: a new anti-tumour approach. *Eur J Surg Oncol* **22**: 152–157

© 2000 Cancer Research Campaign 

Mayer LD, Tai LCL, Ko DSC, Masin D, Ginsberg RS, Cullis PR and Bally MB (1989) Influence of vesicle size, lipid composition, and drug-to-lipid ratio on the biological activity of liposomal doxorubicin in mice. *Cancer Res* **49**: 5922–5930

Murdter TE, Sperker B, Kivisto KT, McClellan M, Fritz P, Friedel G, Linder A, Bosslet K, Toomes H, Dierkesmann R and Kroemer HK (1997) Enhanced uptake of doxorubicin into bronchial carcinoma: beta-glucuronidase mediates release of doxorubicin from a glucuronide prodrug (HMR 1826) at the tumor site. *Cancer Res* **57**: 2440–2445

Nooijen PTGA, Manusama ER, Eggermont AMM, Schalkwijk L, Stavast J, Marquet RL, De Waal RM and Ruiter DJ (1996) Synergistic effects of TNF-alpha and melphalan in an isolated limb perfusion model of rat sarcoma: a histopathological, immunohistochemical and electron microscopical study. *Br J Cancer* **74**: 1908–1915

Olieman AFT, van Ginkel RJ, Hoekstra HJ, Mooyaart EL, Molenaar WM and Koops HS (1997) Angiographic response of locally advanced soft-tissue sarcoma following hyperthermic isolated limb perfusion with tumor necrosis factor. *Ann Surg Oncol* **4**: 64–69

Posner MC, Lienard D, Lejeune FJ, Rosenfelder D and Kirkwood JM (1994) Hyperthermic isolated limb perfusion (HILP) with tumor necrosis factor (TNF) alone for metastatic intransit melanoma. *Proc Am Soc Clin Oncol* **13**: 1351 (abstract)

Prewitt TW, Matthews W, Chaudhri G, Pogrebniak HW and Pass HI (1994) Tumor necrosis factor induces doxorubicin resistance to lung cancer cells in vitro. *J Thorac Cardiovasc Surg* **107**: 43–49

Renard N, Nooijen PT, Schalkwijk L, De Waal RM, Eggermont AM, Lienar, D, Kroon BB, Lejeune FJ and Ruiter DJ (1995) VWF release and platelet aggregation in human melanoma after perfusion with TNF alpha. *J Pathol* **176**: 279–287

Ridge JA, Collin C, Bading JR, Hancock C, Conti PS, Daly JM and Raaf JH (1988) Increased adriamycin levels in hepatic implants of rabbit Vx-2 carcinoma from regional infusion. *Cancer Res* **48**: 4584–4587

Rossi CR, Vecchiato A, Da Pian PP, Nitti D, Lise M, Melanotte PL, Turra S and Vigliani F (1992) Adriamycin in hyperthermic perfusion for advanced limb sarcomas. *Ann Oncol* **3**: S111–113

Ruegg C, Yilmaz A, Bieler G, Bamat J, Chaubert P and Lejeune FJ (1998) Evidence for the involvement of endothelial cell integrin alphaVbeta3 in the disruption of the tumor vasculature induced by TNF and IFN-gamma. *Nat Med* **4**: 408–414

Safrit JT, Berek JS and Bonavida B (1993) Sensitivity of drug-resistant human ovarian tumor cell lines to combined effects of tumor necrosis factor (TNF-alpha) and doxorubicin: failure of the combination to modulate the MDR phenotype. *Gynecol Oncol* **48**: 214–220

Sato N, Goto T, Haranaka K, Satomi N, Nariuchi H, Mano-Hirano YX and Sawasaki Y (1986) Actions of tumor necrosis factor on cultured vascular endothelial cells: morphologic modulation, growth inhibition, and cytotoxicity. *J Natl Cancer Inst* **76**: 1113–1121

Shimomura K, Manda T, Mukumoto S, Kobayashi K, Nakano K and Mori J (1988) Recombinant human tumor necrosis factor-alpha: thrombus formation is a cause of anti-tumor activity. *Int J Cancer* **41**: 243–247

Smyth MJ, Pietersz GA and McKenzie IF (1988) Increased antitumor effect of immunoconjugates and tumor necrosis factor in vivo. *Cancer Res* **48**: 3607–3612

Soranzo C, Perego P and Zunino F (1990) Effect of tumor necrosis factor on human tumor cell lines sensitive and resistant to cytotoxic drugs, and its interaction with chemotherapeutic agents. *Anticancer Drugs* **1**: 157–163

Suzuki S, Ohta S, Takashio K, Nitanai H and Hashimoto Y (1990) Augmentation for intratumoral accumulation and anti-tumor activity of liposome-encapsulated adriamycin by tumor necrosis factor-alpha in mice. *Int J Cancer* **46**: 1095–1100

Tonak J, Hermanek P, Banz H and Groitl H (1979) Cytotoxics and hyperthermic perfusion: a preliminary study. *Cancer Treat Rev* **6**: 135–141

Watanabe N, Niitsu Y, Umeno H, Kuriyama H, Neda H, Yamauchi N, Maeda M and Urushizaki I (1988) Toxic effect of tumor necrosis factor on tumor vasculature in mice. *Cancer Res* **48**: 2179–2183

Weksler B, Lenert J, Ng B and Burt M (1994) Isolated single lung perfusion with doxorubicin is effective in eradicating soft tissue sarcoma lung metastases in a rat model. *J Thorac Cardiovasc Surg* **107**: 50–54

 *© 2000 Cancer Research Campaign*
